# Exogenous Melatonin Enhances Rice Blast Disease Resistance by Promoting Seedling Growth and Antioxidant Defense in Rice

**DOI:** 10.3390/ijms26031171

**Published:** 2025-01-29

**Authors:** Hongliang Yuan, Jingya Qian, Chunwei Wang, Weixi Shi, Huiling Chang, Haojie Yin, Yulin Xiao, Yue Wang, Qiang Li

**Affiliations:** 1 Department of Agronomy, College of Agronomy, Hunan Agricultural University, Changsha 410128, China; yinhaojie@stu.hunau.edu.cn (H.Y.); chunwei@stu.hunau.edu.cn (C.W.); shiweixi@stu.hunau.edu.cn (W.S.); changhuilin@stu.hunau.edu.cn (H.C.); yhl5455@stu.hunau.edu.cn (H.Y.); xiaoyulin@stu.hunau.edu.cn (Y.X.); 2 The Key Laboratory of Crop Germplasm Innovation and Resource Utilization of Hunan Province, Hunan Agricultural University, Changsha 410128, China; qianjingya@stu.hunau.edu.cn; 3 Yuelu Mountain Laboratory of Hunan Province, Hunan Agricultural University, Changsha 410128, China

**Keywords:** rice, melatonin, rice blast, antioxidant enzymes

## Abstract

In order to analyze the physiological regulation mechanisms associated with exogenous melatonin on rice blast, this study treated rice seedlings with different concentrations of melatonin (0, 20, 100, and 500 µmol/L) in order to investigate the growth characteristics, root morphology, superoxide dismutase (SOD) activity, peroxidase (POD) activity, catalase (CAT) activity, malondialdehyde (MDA) content, hydrogen peroxide (H_2_O_2_) content, and soluble protein content of rice seedlings. The results indicated that 100 µmol/L of melatonin exhibited a significant effect, improving the growth and antioxidant capacity of rice seedlings under rice blast fungus infection. The disease resistance level of rice seedlings against rice blast significantly decreased by 31.58% when compared to the 0 µmol/L melatonin treatment, while the plant height, stem base width, plant leaf area, total root length, aboveground dry weight, aboveground fresh weight, and underground fresh weight significantly increased by 8.72% to 91.38%. Treatment with 100 µmol/L of melatonin significantly increased catalase activities and soluble protein content, with respective increases of 94.99% and 31.14%. Simultaneously, the contents of malondialdehyde and hydrogen peroxide significantly decreased, reaching 18.65% and 38.87%, respectively. The gray relational grade analysis indicated that hydrogen peroxide content and resistance level exhibit the highest gray relational grades with melatonin concentration and, so, can be used to evaluate the effect of melatonin on the severity of rice blast fungus infection. Furthermore, the membership function analysis revealed that the 100 µmol/L melatonin treatment had the highest membership function value, indicating a significant improvement in the resistance of rice seedlings to rice blast disease. In conclusion, 100 µmol/L of melatonin enhances the resistance of rice seedlings to rice blast disease through promoting their growth and strengthening their antioxidant defenses. This study provides new insights into the tolerance mechanisms of rice seedlings against rice blast disease.

## 1. Introduction

Rice is the most widely cultivated food crop globally and plays a crucial role in ensuring global food security. By 2050, global food production is expected to increase by 70% to meet the rising demand driven by population growth [1]. At present, enhancing the total yield of rice can be achieved by expanding the planting area and productivity of rice or by minimizing yield losses. However, due to limitations on expanding cultivated land area and irrigation water shortages, further increases in planting area are not feasible. Therefore, mitigating losses associated with adverse environmental factors and reducing post-harvest losses are the only viable strategies to enhance the productivity and overall yield of rice [2]. It is well recognized that rice production is significantly constrained by various biotic and abiotic factors [3].

In terms of biological stresses, rice blast (*Maganporte oryzae*) represents the most significant constraint on global rice yield, directly reducing rice yields and indirectly increasing production costs [4]. Rice blast disease occurs at various stages of rice development, primarily affecting the leaves, stems, and panicles, and causes significant yield reductions. In severe cases, it can lead to a 40% to 50% reduction in yield or even complete crop failure, posing a serious threat to global rice production safety [5]. Thus, in order to enhance rice yields and overcome the challenges posed by rice blast disease, various control methods are being developed.

Previous studies have demonstrated that chemical control and the cultivation of disease-resistant varieties allow for the effective prevention and management of rice blast disease. However, the complexity and variability of rice blast fungus inheritance mean that its virulence will continue to evolve, leading to the potential loss of rice blast resistance in bred varieties. Additionally, cultivating rice blast-resistant varieties is often challenging and time-consuming [6,7]. Although synthetic fungicides are typically effective in controlling plant diseases, as the demand for agricultural production increases, the overuse of fungicides may not only exacerbate negative environmental impacts but also poses risks to human health [8,9]. Furthermore, the widespread application of fungicides may also increase the likelihood of developing pathogen-resistant populations [10]. Thus, it is essential to identify and develop an eco-friendly approach, which is capable of efficiently controlling rice blast, either to replace or to be used in rotation with existing fungicides in order to achieve better rice blast management. Research has shown that enhancing the resistance of susceptible rice varieties to rice blast is a cost-effective and environmentally sustainable method for improving both yield and quality [11,12].

Plant hormones play a crucial role in enhancing the tolerance and adaptability of plants under environmental stresses [13]. Melatonin (N-acetyl-5-methoxytryptamine) is a vital and multifunctional signaling molecule which is found abundantly in various plant species [14]. Melatonin was first discovered in plants in 1995 [15,16] and can regulate various physiological and stress-resistance processes in plants. As such, it plays a crucial role in plant processes such as germination, photosynthesis, circadian rhythms, flowering, senescence, and responses to adverse environmental conditions [17,18,19]. To date, the application of exogenous melatonin to enhance the tolerance of plants to challenging environments has been widely demonstrated. Previous studies have shown that melatonin treatment significantly enhances the salt and drought tolerance of rice plants through increasing biomass, inhibiting ROS accumulation, boosting antioxidant defense efficiency, and upregulating the expression of genes involved in salt and drought stress responses [20]. Han et al. also reported that the application of exogenous melatonin can alleviate the inhibitory effects of low-temperature stress on rice growth through enhancing the activity of antioxidant enzymes and the levels of non-enzymatic antioxidants in rice [21]. Cui et al.’s study showed that melatonin treatment can significantly enhance the antioxidant capacity of wheat seedlings, reduce endogenous ROS levels, alleviate membrane damage, and thus significantly improve the drought tolerance of wheat seedlings [22]. Yan et al. studied the effect of melatonin on the antioxidant capacity of rice under salt stress. It was found that melatonin reduced the inhibitory effect of salt stress on rice seedling growth by increasing the dry and fresh weight of aboveground and underground parts [23]. Melatonin has also been widely applied to alleviate the toxic effects of metal ions on plants. For instance, Jiang et al. found that exogenous melatonin improved rice grain yield and reduced heavy metal accumulation through regulating the plant’s antioxidant capacity and metabolic processes, as well as altering the physicochemical properties of the soil and the structure of the microbial community [24]. Raheel Munir et al. conducted an in-depth study on the mechanisms associated with exogenous melatonin application in rice under cadmium stress and found that melatonin exhibited a correlation with stress response plant hormones (e.g., ABA and IAA). It enhanced the plant’s defense mechanism by increasing the activities of ROS-scavenging antioxidant enzymes (e.g., SOD, POD, CAT, and APX) and regulating key stress response genes, thereby restoring cell membrane integrity and increasing tolerance to cadmium toxicity [25]. Moreover, treatment with melatonin not only enhances the plant’s resistance to a variety of pathogens, including bacteria, fungi, and viruses, but also effectively regulates the severity of broad-spectrum pathogens and diseases within the plant [26]. Chen et al. found that spraying exogenous melatonin enhanced the resistance of rice to bacterial blight through inhibiting bacterial division and proliferation by reducing cell division and decreasing the activity of enzymes involved in metabolism [27,28]. Lu et al. demonstrated that melatonin treatment enhanced the resistance of rice to rice stripe virus (RSV) through inducing the nitric oxide (NO) signaling pathway [29]. Kuberan Thangaraj et al. found that melatonin could also enhance the resistance of tea plants to sooty mold by promoting the activities of antioxidant enzymes in the plant [30]. Foliar application of melatonin enhanced the resistance of eggplant to alfalfa mosaic virus infection by promoting the accumulation of phenolic and flavonoid compounds, in addition to activating the antioxidant defense system, gene expression, and molecules involved in scavenging free radicals [31].

Previous studies have found that the application of exogenous melatonin can promote plant growth and development under both biotic and abiotic stresses. However, there are limited reports on the physiological regulatory mechanisms of exogenous melatonin in rice seedlings infected with the rice blast fungus (*Magnaporthe oryzae*). Therefore, in order to further explore the mechanism of exogenous melatonin treatment in controlling rice blast disease and study its effects on the growth and antioxidant capacity of rice seedlings, this experiment utilized the rice variety Songyazao 1 (SYZ1) in order to investigate the effects of different concentrations of melatonin on the growth characteristics, root morphology, and physiological traits of rice seedlings infected with the rice blast fungus. This study aims to explore the physiological regulatory mechanisms of melatonin in rice seedlings under rice blast infection.

## 2. Results

### 2.1. Evaluation of Exogenous Melatonin in Enhancing Rice Resistance to Blast Disease

**Figure 1:**
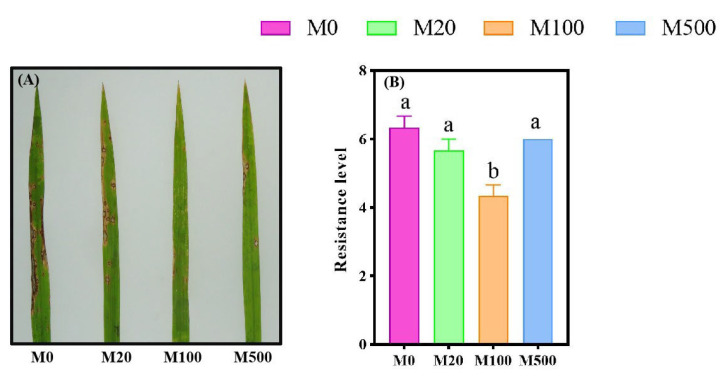
The effects of exogenous melatonin on the resistance levels of rice seedlings against blast disease under pathogen infection. Means ± SEs with different letters in each parameter indicate significant statistical differences (*p* < 0.05). (**A**): The disease phenotype; (**B**): resistance level.

Exogenous melatonin has a certain promoting effect, enhancing the resistance of rice seedlings to rice blast disease (Figure 1). Among the treatments, the M100 melatonin application exhibited the most significant effect. Compared with the M0, M20, and M500 treatments, the resistance levels of rice seedlings to rice blast disease were significantly reduced by 31.58%, 23.53%, and 27.78%, respectively.

### 2.2. Effects of Exogenous Melatonin on Rice Biomass

**Figure 2:**
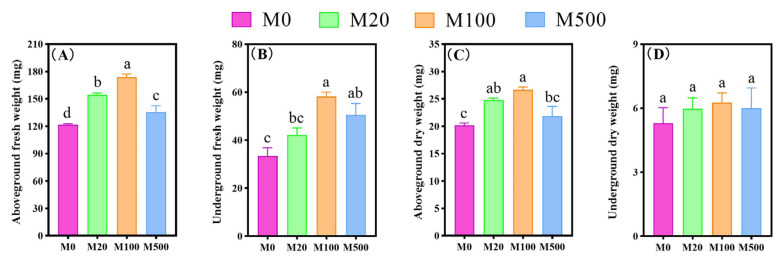
The effects of exogenous melatonin on the dry and fresh weights of rice seedlings under blast fungus infection. Means ± SEs with different letters in each parameter indicate significant statistical differences (*p* < 0.05). (**A**): Aboveground fresh weight; (**B**): underground fresh weight; (**C**): aboveground dry weight; (**D**): underground dry weight.

Exogenous melatonin treatment exhibited a certain promoting effect on the fresh and dry weight of the aboveground and underground parts of rice seedlings infected with the rice blast fungus (Figure 2). Specifically, compared with the M0 treatment, the M20 treatment increased the fresh and dry weight of the aboveground parts of rice seedlings by 26.93% and 22.64%, respectively. The M100 treatment increased the aboveground fresh weight, aboveground dry weight, and underground fresh weight of rice seedlings by 42.9%, 32.07%, and 74.5%, respectively. The M500 treatment increased the fresh weight of the aboveground and underground parts of rice seedlings by 11.32% and 51.5%, respectively.

**Figure 3:**
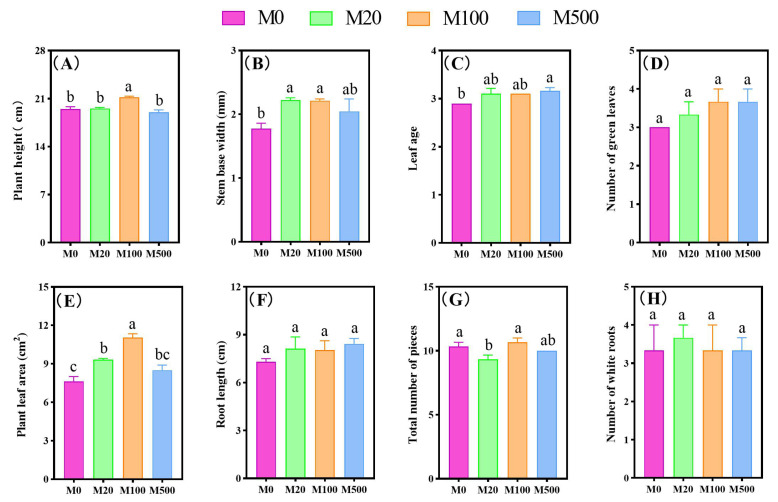
The effects of exogenous melatonin on the growth characteristics of rice seedlings under blast fungus infection. Means ± SEs with different letters in each parameter indicate significant statistical differences (*p* < 0.05). (**A**): Plant height; (**B**): stem base width; (**C**): leaf age; (**D**): number of green leaves; (**E**): plant leaf area; (**F**): root length; (**G**): total number of pieces; (**H**): number of white roots.

Exogenous melatonin treatment alleviated the inhibitory effect of rice blast fungus infection on rice growth (Figure 3). The M20 treatment significantly increased the stem base width and plant leaf area of rice seedlings, with increases of 25.38% and 22.16%, respectively, compared to the M0 treatment. The M100 treatment significantly increased the plant height, stem base width, and plant leaf area of rice seedlings, with increases of 8.72%, 24.81%, and 44.75%, respectively, compared to the M0 treatment. The M500 treatment significantly increased the leaf age of rice seedlings by 9.2% compared to the M0 treatment.

### 2.3. Effects of Exogenous Melatonin on Rice Root Morphology

**Figure 4:**
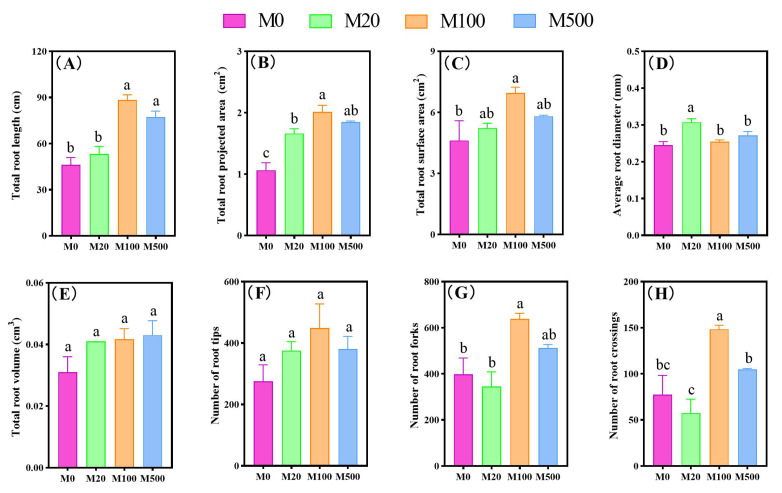
The effects of exogenous melatonin on the root morphology of rice seedlings under blast fungus infection. Means ± SEs with different letters in each parameter indicate significant statistical differences (*p* < 0.05). (**A**): Total root length; (**B**): total root projected area; (**C**): total root surface area; (**D**): average root diameter; (**E**): total root volume; (**F**): number of root tips; (**G**): number of root forks; (**H**): number of root crossings.

Under the rice blast fungus infection, exogenous melatonin treatment exhibited a promoting effect on the root morphology of rice seedlings (Figure 4). Specifically, the total root projected area and average root diameter of rice seedlings treated with M20 were significantly increased by 56.4% and 25.52%, respectively, compared to the M0 treatment. The total root length, total root projected area, total root surface area, number of root forks, and number of root crossings of rice seedlings treated with M100 were significantly increased by 50.43% to 91.81%, compared to the M0 treatment. The total root length and total root projected area of rice seedlings treated with M500 were significantly increased by 67.17% and 73.77%, respectively, compared to the M0 treatment.

### 2.4. Effects of Exogenous Melatonin on Antioxidant Enzyme Activity in Rice

**Figure 5:**
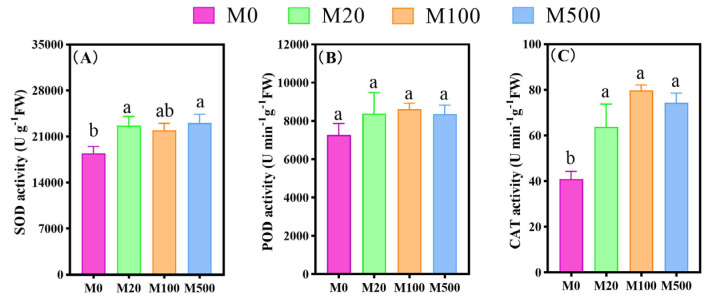
The effects of exogenous melatonin on the activities of antioxidant enzymes in rice seedlings under blast fungus infection. Means ± SEs with different letters in each parameter indicate significant statistical differences (*p* < 0.05). (**A**): SOD activity; (**B**): POD activity; (**C**): CAT activity.

Exogenous melatonin treatment enhanced the antioxidant capacity of rice seedlings infected with rice blast fungus (Figure 5). Among them, the SOD and CAT activities of rice seedlings treated with M20 were significantly increased by 22.74% and 55.83%, respectively, compared to the M0 treatment. The CAT activity of rice seedlings treated with M100 significantly increased by 94.99%, compared to the M0 treatment. The SOD and CAT activities of rice seedlings treated with M500 were significantly increased by 24.89% and 81.6%, respectively, compared to the M0 treatment.

### 2.5. Effects of Exogenous Melatonin on MDA Content, H₂O₂ Content, and Soluble Protein Content in Rice

**Figure 6:**
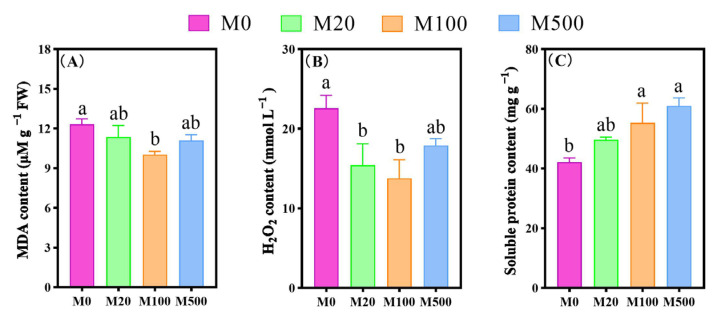
The effects of exogenous melatonin on MDA, H_2_O_2_, and soluble protein contents in rice seedlings under blast fungus infection. Means ± SEs with different letters in each parameter indicate significant statistical differences (*p* < 0.05). (**A**): MDA content; (**B**): H_2_O_2_ content; (**C**): soluble protein content.

Exogenous melatonin treatment mitigated cell damage and oxidative stress in rice seedlings infected with rice blast fungus (Figure 6). Specifically, the MDA content of rice seedlings treated with M100 was significantly reduced by 18.65%, compared to the M0 treatment. The H₂O₂ contents in rice seedlings treated with M20 and M100 were significantly decreased by 31.62% and 38.87%, respectively, compared to the M0 treatment. Additionally, the soluble protein contents of rice seedlings treated with M100 and M500 were significantly increased by 31.14% and 44.67%, respectively, compared to the M0 treatment.

### 2.6. Gray Relational Analysis of Exogenous Melatonin on Rice Seedling Growth under Blast Fungus Infection

**Figure 7:**
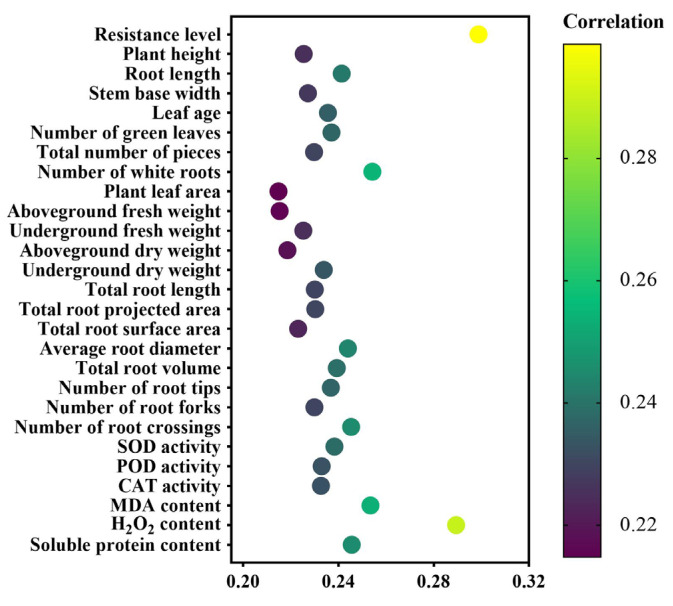
Gray relational grade analysis of exogenous melatonin on growth and physiological indices of rice seedlings under rice blast fungus infection.

Through gray relational grade analysis between the physiological indices of rice seedlings infected with blast fungus and melatonin concentrations, as expected, it was found that resistance level and H_2_O_2_ content exhibited the highest gray relational grades with melatonin concentration (Figure 7). Therefore, these indicators can be used to evaluate the alleviating effect of melatonin on the growth of rice seedlings under rice blast fungus infection. Notably, the gray relational grade between resistance level and melatonin concentration was the highest, indicating that resistance level is the most reliable indicator for assessing the effectiveness of melatonin in mitigating rice blast fungus infection. In addition, the content of H_2_O_2_ can also effectively reflect the alleviation effect of melatonin on the growth of rice seedlings under rice blast fungus infection.

### 2.7. Correlation Analysis of Exogenous Melatonin with Various Indices of Rice Seedlings under Blast Fungus Infection

**Figure 8:**
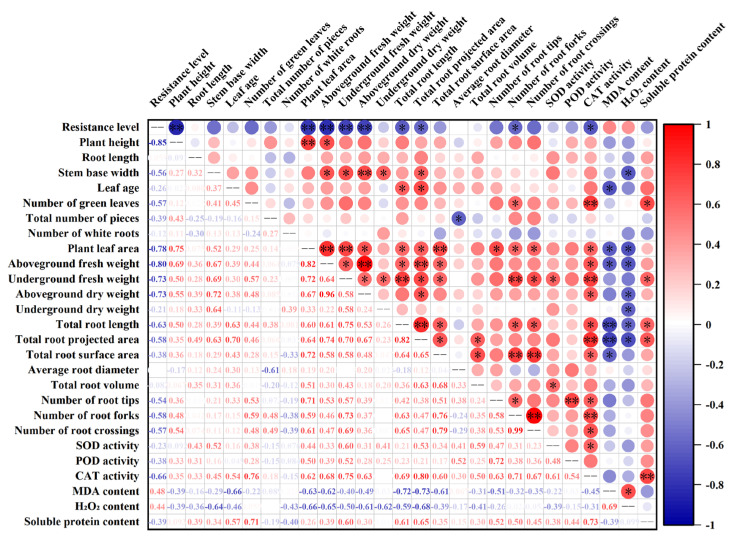
Correlation analysis of exogenous melatonin with various growth indicators of rice seedlings infected with rice blast fungus; **: significant at the 0.01 probability level (*p* < 0.01); *: significant at the 0.05 probability level (*p* < 0.05).

As illustrated in Figure 8, the correlation analysis of exogenous melatonin with various indicators of rice seedling growth under rice blast fungus infection showed that the resistance level of rice seedling leaves was significantly negatively correlated with plant height, plant leaf area, aboveground fresh weight, underground fresh weight, and aboveground dry weight (*p* < 0.01), and significantly negatively correlated with total root length, total root projected area, number of root forks, and CAT activity (*p* < 0.05). The plant leaf area of rice seedlings was significantly positively correlated with plant height, aboveground fresh weight, underground fresh weight, and total root surface area (*p* < 0.01). CAT activity showed highly significant positive correlations (*p* < 0.01) with soluble protein content, number of green leaves, underground fresh weight, total root projected area, and number of root forks. MDA content was significantly negatively correlated with total root length and total root projected area (*p* < 0.01), and H_2_O_2_ content was significantly negatively correlated with stem base width, plant leaf area, aboveground fresh weight, aboveground dry weight, total root length, and total root projected area (*p* < 0.05), and significantly positively correlated with MDA content (*p* < 0.05).

### 2.8. Principal Component Analysis of the Effects of Exogenous Melatonin on Various Indicators in Rice Seedlings Infected with Rice Blast Fungus

**Figure 9:**
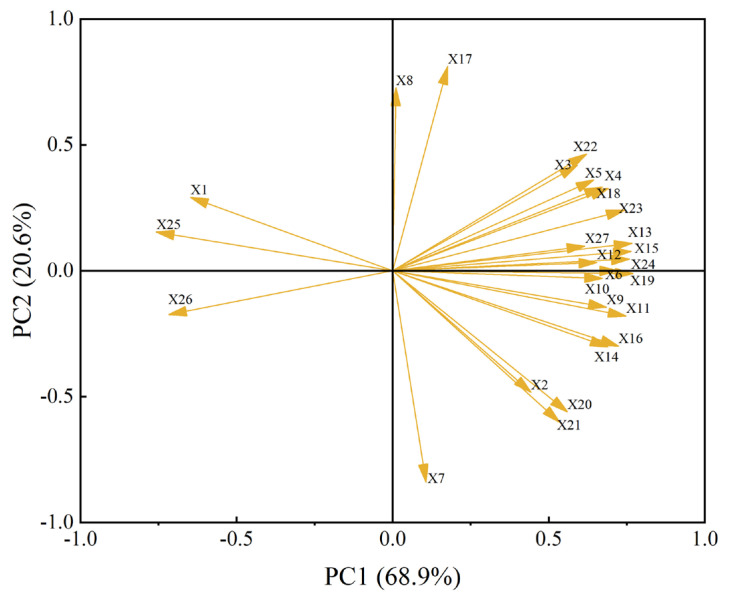
Principal component analysis of exogenous melatonin on various indicators of rice seedlings infected with rice blast fungus. X1: Resistance level; X2: plant height; X3: root length; X4: stem base width; X5: leaf age; X6: number of green leaves; X7: total number of pieces; X8: number of white roots; X9: plant leaf area; X10: aboveground fresh weight; X11: underground fresh weight; X12: aboveground dry weight; X13: underground dry weight; X14: total root length; X15: total root projected area; X16: total root surface area; X17: average root diameter; X18: total root volume; X19: number of root tips; X20: number of root forks; X21: number of root crossings; X22: SOD activity; X23: POD activity; X24: CAT activity; X25: MDA content; X26: H_2_O_2_ content; X27: soluble protein content.

Principal component analysis (PCA) is a method for simplifying the structure of data through linearly combining multiple original variables into a smaller number of composite indicators. These new comprehensive indicators not only effectively reflect the main information of the original variables but also ensure that there are no inter-relations between the different indicators, thereby achieving more accurate data analysis results [32]. In this study, principal component analysis was employed to analyze the 27 indicators of rice seedling response under rice blast fungus infection (Figure 9). The cumulative contribution rate of the first two principal components (PCA1 and PCA2) was found to be 89.46%, indicating that these components integrate the majority of information related to rice seedling growth, which meets the requirements of principal component analysis. Among them, the characteristic value of PCA1 was 18.6, with a contribution rate of 68.89%. Positive characteristic values associated with PCA1 include the number of root tips, underground dry weight, total root projected area, and CAT activity, while negative characteristic values with larger absolute values include MDA content, H_2_O_2_ content, and resistance level. The characteristic value of PCA2 was 5.55, with a contribution rate of 20.57%. Positive characteristic values associated with PCA2 include average root diameter, number of white roots, SOD activity, and root length, while negative characteristic values with larger absolute values include the total number of pieces, number of root crossings, and plant height.

### 2.9. Membership Function Analysis of Various Indicators in Rice Seedlings Infected with Rice Blast Fungus Treated with Exogenous Melatonin

**Figure 10:**
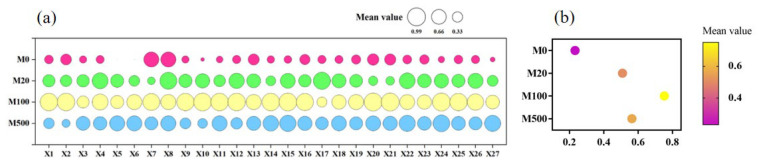
Membership function analysis of various indicators in rice seedlings infected with rice blast fungus and treated with exogenous melatonin. (**a**): value of the membership function; (**b**): mean value of the membership function; X1: Resistance level; X2: plant height; X3: root length; X4: stem base width; X5: leaf age; X6: number of green leaves; X7: total number of pieces; X8: number of white roots; X9: plant leaf area; X10: aboveground fresh weight; X11: underground fresh weight; X12: aboveground dry weight; X13: underground dry weight; X14: total root length; X15: total root projected area; X16: total root surface area; X17: average root diameter; X18: total root volume; X19: number of root tips; X20: number of root forks; X21: number of root crossings; X22: SOD activity; X23: POD activity; X24: CAT activity; X25: MDA content; X26: H_2_O_2_ content; X27: soluble protein content.

Under infection with rice blast fungus, the various indicators of rice seedling growth presented different responses to the exogenous melatonin treatments. Therefore, the membership function method was used to comprehensively evaluate the various indicators of rice seedlings treated with melatonin (Figure 10). The results indicated that the membership function value of the M100 treatment was the highest, indicating that M100 had the most significant effect in enhancing the resistance of rice seedlings to rice blast disease, when compared to the M20 and M500 treatments.

## 3. Discussion

Rice blast is a devastating fungal disease of rice, imperiling global food security. Melatonin, as a novel plant growth regulator, is believed to be involved in various biotic and abiotic stress responses.

Among plant hormones, melatonin has garnered significant attention due to its beneficial properties for plants. Previous studies have demonstrated that exogenous melatonin significantly reduced the incidence of potato late blight by inhibiting the growth of *Phytophthora infestans hyphae*, altering the cellular ultrastructure, reducing the virulence of the pathogen, and diminishing its resistance to stress [33]. In addition, melatonin enhanced the resistance of cucumber plants to fusarium wilt disease by promoting their photosynthetic capacity, antioxidant defense, and secondary metabolite contents [34]. Mandal M.K. et al. found that increased melatonin levels in plants boosted resistance against the foliar pathogen *Podosphaera xanthii* (powdery mildew) and the soil-borne oomycete *Phytophthora capsici* in watermelon and other cucurbits [35]. In this study, exogenous melatonin was found to effectively enhance the resistance of rice seedlings to rice blast disease. Among them, the M100 treatment had the most significant effect, with a significant reduction of 31.58% in the resistance level of rice seedlings compared to the control. In this study, we found that, under the rice blast fungus infection, exogenous melatonin significantly improved the growth and development of rice seedlings and had positive effects on plant height, stem base width, plant leaf area, and the underground and aboveground biomass of rice seedlings, with the M100 treatment presenting the most significant effect. Similarly, Muhammad Imran et al. investigated the effects of exogenous melatonin application (foliar or root zone) on improving drought tolerance in soybean seedlings. They found that applying melatonin at a concentration of 100 µmol/L significantly increased the chlorophyll content, as well as the root and stem length and biomass of soybean plants [36]. Melatonin has been shown to enhance crop stress resistance by promoting root growth. Previous studies have indicated that melatonin treatment had a significant impact on the root structure of rice, including the total root length, total root projection area, total root surface area, number of root tips, and the number of root crossings [37]. The research results of Huang et al. revealed that the application of melatonin significantly improved the root vitality of tomato seedlings, significantly increasing root morphology parameters (e.g., total root length, total root surface area, average root diameter, and lateral root number), improving root morphology and enhancing the resistance of tomato seedlings to drought stress [38]. Similarly, applying 100 µmol/L of melatonin resulted in denser and longer roots in kiwifruit under drought stress, thereby alleviating the adverse effects of drought stress on kiwifruit seedling growth [39]. In this study, melatonin treatment significantly promoted root growth in rice seedlings, with the application of 100 µmol/L of melatonin notably increasing the total root length, total root projected area, total root surface area, number of root forks, and number of root crossings by 91.38%, 89.3%, 50.43%, 60.55%, and 91.81%, respectively. These findings suggest that melatonin enhanced the growth of both the aboveground and underground parts of the plant.

Rice may encounter a variety of biotic and abiotic stress challenges throughout its growth and development stages. Reactive oxygen species (ROS) are normal byproducts of plant cell metabolism and primarily include hydrogen peroxide (H₂O₂), superoxide (O₂⁻), and hydroxyl radicals (·OH) [40]. The excessive accumulation of ROS leads to redox imbalance and disrupts signal transduction processes, resulting in cell damage and growth inhibition. Plant antioxidant enzyme systems, such as superoxide dismutase (SOD), peroxidase (POD), catalase (CAT), and ascorbate peroxidase (APX), play a crucial role in eliminating ROS and mitigating oxidative stress, thereby protecting the cells from damage [41]. Previous studies have shown that melatonin is an effective ROS scavenger and a potent endogenous free radical scavenger. For instance, the foliar application of 100 µmol/L of melatonin under drought stress increased the survival rate of rice seedlings, strengthened their antioxidant systems, and regulated osmotic balance, thereby significantly mitigating the damage caused by drought stress in rice seedlings [42]. Similarly, studies on exogenous melatonin application in white bean seedlings under salt stress have shown that increasing melatonin concentration was positively correlated with the plant’s stress tolerance. Specifically, 100 µmol/L of melatonin demonstrated significant effects in terms of improving growth parameters and enhancing the antioxidant capacity [43]. Superoxide dismutase (SOD) serves as the first line of defense against ROS toxicity through catalyzing the dismutation of superoxide anion (O₂⁻) into hydrogen peroxide (H₂O₂). Catalase (CAT), which has the highest conversion efficiency among antioxidant enzymes, subsequently breaks down H₂O₂ into water and oxygen. The synergistic action of these two enzymes effectively reduces the accumulation of ROS. Peroxidase (POD) catalyzes the oxidation of various substrates in the presence of H₂O₂, working in coordination with SOD and CAT to eliminate excess free radicals. In this study, melatonin effectively enhanced the antioxidant defense system of rice seedlings by increasing the activities of key antioxidant enzymes such as SOD and CAT. The excessive accumulation of ROS can cause oxidative damage to cells, leading to the lipid peroxidation of cell membranes, which in turn affects the normal metabolic functions of plants. Malondialdehyde (MDA) is an indicator of membrane lipid peroxidation, and the decrease in its content indicated that melatonin treatment reduced oxidative damage in rice seedlings. In addition, melatonin treatment also promoted the accumulation of soluble proteins, which act as osmotic regulators in plant metabolism, helping to alleviate and reduce the damage caused by stress conditions such as drought. On the other hand, melatonin treatment positively influenced ROS scavenging by reducing the accumulation of H₂O₂. While H₂O₂—a typical ROS—participates in signal transduction at low concentrations, excessive levels can trigger oxidative stress, thus damaging cellular structures and functions. Melatonin effectively decomposes H₂O₂ through enhancing CAT activity, thereby reducing its potential threat to cells. This action not only alleviated the oxidative damage caused by rice blast fungus infection, but likely also promoted metabolic balance and the recovery ability of plant cells. In summary, melatonin significantly enhanced disease resistance and growth characteristics in rice seedlings by increasing the activities of antioxidant enzymes, reducing ROS accumulation, and promoting the accumulation of soluble proteins. It is worth noting that a concentration of 100 µmol/L of melatonin demonstrated significant effects across various indicators, suggesting it as the optimal treatment concentration. Future studies should further explore the effects of different melatonin concentrations at various growth stages and under diverse environmental conditions, aiming to provide a more comprehensive theoretical basis for disease prevention and growth regulation in rice.

Gray relational grade analysis is a comprehensive evaluation method based on gray system theory and utilizing multiple factors, which can objectively evaluate the degree of correlation between various factors within the system; simultaneously, its results have strong reliability [44]. However, there are currently few reports on the application of gray relational analysis in the context of rice blast resistance. This study used gray relational analysis to comprehensively evaluate the effects of melatonin on the physiological indicators of rice seedlings. The results showed that the gray correlations between H_2_O_2_ content, resistance level, and MDA content with melatonin concentration were the highest and most closely related. These results can serve as indicators for measuring the alleviating effect of melatonin on the growth of rice seedlings infected with rice blast fungus. MDA serves as an indicator of membrane lipid peroxidation, with its increase reflecting oxidative damage to the cellular defense system under stress conditions. In this experiment, after melatonin application, the H_2_O_2_ content, MDA content, and resistance level in rice seedlings were significantly reduced. This indicates that, under rice blast fungus infection, decreasing the H_2_O_2_ and MDA levels in rice seedlings can further promote seedling growth, thereby enhancing their resistance to rice blast and lowering their resistance level. The correlation analysis results demonstrated a significant positive correlation between H_2_O_2_ and MDA contents, suggesting that reducing H_2_O_2_ levels can effectively mitigate oxidative damage to the cellular defense system in rice seedlings under rice blast fungus infection.

**Figure 11:**
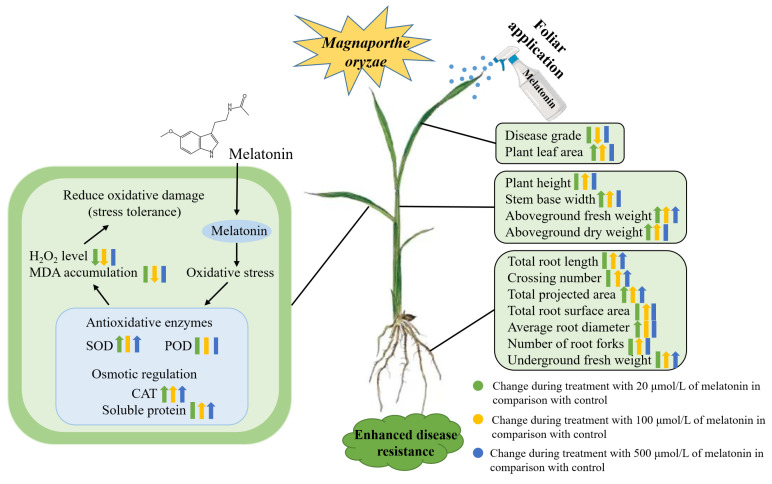
Exogenous melatonin enhances the physiological mechanisms of rice blast resistance by promoting the growth and antioxidant defense of rice seedlings. The symbols (↑), (↓), and (|) represent upregulation, downregulation, and no significant changes for various parameters, respectively.

Due to the inability of single-indicator analysis to accurately evaluate the growth of rice seedlings, the use of principal component and membership function analyses for comprehensive plant evaluation enhanced the objectivity and accuracy of the obtained results. This approach mitigates the biases associated with single indicators by integrating multiple measurements for a more holistic assessment [45]. Our research findings indicate that exogenous melatonin treatment can enhance the resistance of rice to rice blast disease. Through comparing average membership function values, we found that 100 µmol/L melatonin had the most pronounced promoting effect on rice. This study explored the mechanisms through which melatonin alleviates the effects of rice blast fungus infection on the growth and development of rice seedlings for the first time. The physiological mechanism of 100 µmol/L melatonin promoting the growth and development of rice seedlings infected with rice blast fungus is shown in Figure 11. These insights may provide a foundation for future innovations in regulating plant disease resistance through the use of melatonin.

## 4. Materials and Methods

### 4.1. Test Material

The tested susceptible rice variety was SYZ1, which is an indica conventional rice variety that is widely cultivated in the middle and lower reaches of the Yangtze River in China. It has a complete growth cycle of 110.8 days and was provided by the Hunan Rice Research Institute. Melatonin (N-acetyl-5-methoxytryptamine, MT) was obtained from Sigma-Aldrich (St. Louis, MO, USA). All chemicals used in this study were of analytical grade.

### 4.2. Experimental Design

Rice seeds with uniform plumpness were selected and disinfected by soaking in a 5% sodium hypochlorite solution for 40 min, followed by thorough rinsing with clean water 4 times. Afterward, the seeds were soaked in clean water for 24 h and then placed in a constant-temperature incubator to germinate. Once germinated, the seeds were sown in seedling trays. The trays were then transferred to an RLD-1000E-4 artificial climate chamber (Ningbo le electrical instrument manufacturer Co., LTD, Ningbo) for treatment, where the temperature was set to 26 °C, the photoperiod to 12 h, and the relative humidity to 70%. At the two-leaf, one-heart stage (on the 15th day after sowing) of the rice seedlings, exogenous melatonin was sprayed at different concentrations; in particular, the melatonin was set at four concentrations: 0 µmol/L, 20 µmol/L, 100 µmol/L, and 500 µmol/L (labeled as M0, M20, M100, and M500, respectively). Subsequently, rice seedlings treated with different concentrations of melatonin were transplanted into the resistance identification nursery for rice blast disease in Liuyang, Changsha, China (28°20′ N and 114°15′ E). Adopting a random block design, each treatment has an area of 0.5 square meters (length and width of 0.5 m and 1 m, respectively). The plant spacing and row spacing of rice plants are 10 cm × 25 cm, respectively. In order to promote the better induction of rice blast disease, four rows of rice variety CO39 were planted around each treatment. This variety is highly susceptible to rice blast disease. When all seedlings treated with melatonin were transplanted into the field, CO39 had already developed symptoms and had obvious gray–white spots, which was considered as the 1st day of infection. Field management prohibits the use of any fungicides. The blast nursery evaluations were performed in April of 2023 in Liuyang Country, Changsha, China, as previously described by Wang et al. [46]. This nursery contains 11 major blast races, including ZC9, ZC11, ZE3, ZB29, ZG1, ZB25, ZB31, ZB13, ZC7, ZA9, and ZF19. On the 15th day after infection with the rice blast fungus, the resistance levels of the rice seedlings to rice blast disease, in relation to the treatments using different concentrations of melatonin, were investigated and recorded. Relevant indicators such as growth characteristics, root morphology, and physiological traits were measured, at each treatment.

### 4.3. Measurement Items and Methods

#### 4.3.1. Rice Blast Resistance Identification

The resistance levels of rice blast and leaf blast were recorded according to the standard for investigation and grading of rice seedling leaf blast (Table 1) [47] (pp. 14–15). For each treatment, ten plants were selected, and the three most severely infected leaves per plant were examined. Disease severity was assessed and recorded for each plant, with a rating scale where scores of 0–3 were classified as resistance type (R), and scores of 4–9 as susceptible type (S). The average disease severity was then calculated for each treatment.

#### 4.3.2. Determination of Biomass in Rice Seedlings

For each treatment, three seedlings were randomly selected, and measurements of plant height, root length, leaf age, number of green leaves, number of white roots, total number of pieces, and stem base width were taken. Plant height is the height from the base of the seedling to the top of the highest leaf. Root length is measured as the length of the primary root. The leaf age is the sum of the fully exposed leaf (denoted as an integer) and the exposed leaf (the ratio of the actual length to the estimated length, denoted as a decimal). The number of green leaves is the number of green leaves per plant. The number of white roots was determined visually, measuring the number of roots that are white from the root to the root tip and have a length of less than 2 cm. The total number of pieces refers to the number of all roots in the root system. Stem base width was measured as the width at the thickest point of the seedling.

For each treatment, three seedlings were randomly selected. After being thoroughly rinsed with tap water and removing excess moisture, the shoots and roots were separated at the junction using scissors, dividing them into aboveground and underground parts. The fresh weights of both the aboveground and underground parts were then measured separately using a 0.0001 g analytical balance (MettlerToledoME104E, Switzerland). The fresh samples were then placed in an oven at 105 °C for 30 min for deactivation, followed by drying at 80 °C until a constant weight was achieved. The dry weights of the samples were then measured using the same 0.0001 g analytical balance.

#### 4.3.3. Determination of Plant Leaf Area

For each treatment, three seedlings were randomly selected. A ruler was employed to measure the length and width of each leaf. The leaf area was calculated using the following formula [48]:

Leaf Area = Leaf Length × Leaf Width × K (K = 0.75)

#### 4.3.4. Determination of Root Morphology

For each treatment, three seedlings were randomly selected. After rinsing with tap water and removing excess moisture, the shoot and root were separated at the junction using scissors. The root morphology was determined using the WinRHIZO root analysis system (Epson Perfection V850 Pro, Beijing, China).

#### 4.3.5. Determination of the Physiological and Biochemical Indicators

The superoxide dismutase (SOD) activity was measured via the nitro blue tetrazolium method [49]; the peroxidase (POD) activity was measured via the guaiacol method [50]; the catalase (CAT) activity was determined via the UV absorption method [51]; the malondialdehyde (MDA) content was determined via the thiobarbituric acid colorimetric method [52]; the soluble protein content was determined via the Coomassie brilliant blue G-250 staining method [53]; and the hydrogen peroxide (H_2_O_2_) content was determined using the Hydrogen Peroxide (H_2_O_2_) Content Assay Kit (Beijing Solarbio Science and Technology Co., Ltd, Beijing, China).

### 4.4. Determination of the Physiological and Biochemical Indicators

The data were organized and analyzed using the Microsoft Excel 2016 software. Data analysis was conducted with the DPS 9.01 software, employing Duncan’s new complex difference method for mean comparison at a significance level of *p* < 0.05. Statistical results were plotted and compared using Origin 2024 and GraphPad Prism 9.3.1.

## 5. Conclusions

This study evaluated the effects of exogenous melatonin treatments on the resistance of rice seedlings to blast disease and explored the associated potential mechanisms. Exogenous melatonin treatment significantly enhanced the antioxidant defense system of rice seedlings. Specifically, the activities of antioxidant enzymes such as superoxide dismutase and catalase were enhanced, while the soluble protein content in rice seedlings was increased. Concurrently, the levels of reactive oxygen species, including malondialdehyde and hydrogen peroxide, were significantly reduced. This phenomenon indicates that melatonin activates the antioxidant defense system of rice seedlings, reduces the accumulation of reactive oxygen species, and effectively alleviates oxidative stress damage in seedlings, ultimately improving their resistance to rice blast disease. Moreover, melatonin also enhanced the growth of rice seedlings, increasing the fresh and dry weight of the aboveground parts and the fresh weight of the underground parts. This further confirms the important role of melatonin in promoting growth and enhancing disease resistance in rice. The results of this study confirm that exogenous melatonin, as a safe and effective plant growth regulator, has the potential to improve the disease resistance and growth development of rice seedlings and can provide an alternative means to enhance their resistance to rice blast. This research provides new insights and possibilities for rice production and may provide a scientific basis for disease prevention and the efficient production of crops such as rice.

## Figures and Tables

**Table 1:** Standard for investigation and grading of rice seedling leaf blast resistance.

Scores	Description	Type
0	No disease lesions	HR
1	Only small brown specks the size of pinpoints	R
2	Larger brown specks	R
3	Slightly larger round gray lesions, brown edge, lesion diameter 1–2 mm	MR
4	Typical spindle-shaped lesions, 1–2 mm long, usually confined between two leaf veins	MR
5	Fusiform lesions, with an affected area of 4–10% of the leaf area	S
6	Fusiform lesions, with an affected area of 11–25% of the leaf area	S
7	Fusiform lesions, with an affected area of 26–50% of the leaf area	S
8	Fusiform lesions, with an affected area of 51–75% of the leaf area	HS
9	Fusiform lesions, with an affected area greater than 75% of the leaf area, or complete leaf death	HS

The high resistance type was identified as HR, the resistance type was identified as R, the medium resistance type was identified as MR, the susceptible type was identified as S, and the highly susceptible type was identified as HS.

## Data Availability

All of the data are contained within the article.
